# Suppression of CRTC2-mediated hepatic gluconeogenesis by TRAF6 contributes to hypoglycemia in septic shock

**DOI:** 10.1038/celldisc.2016.46

**Published:** 2016-12-13

**Authors:** Sihan Lv, Xinchen Qiu, Jian Li, Weida Li, Chao Zhang, Zhen-Ning Zhang, Bing Luan

**Affiliations:** 1 Department of Endocrinology, Shanghai Tenth People’s Hospital, School of Medicine, Tongji University, Shanghai, China; 2 Translational Medical Center for Stem Cell Therapy & Institute for Regenerative Medicine, Shanghai East Hospital, School of Life Sciences and Technology, Tongji University, Shanghai, China

**Keywords:** CRTC, gluconeogenesis, septic shock, TRAF6, ubiquitination

## Abstract

Although hypoglycemia has been documented as a major cause of high mortality in the setting of septic shock, the mechanism of hypoglycemia in infection has not been clearly determined. Hepatic gluconeogenesis serves as an important mechanism to maintain glucose levels under physiological conditions and CREB coactivator CRTC2 plays an important role in regulating gluconeogenic gene expression. Here, we show that triggering of the Toll-like receptor 4 pathway in response to endotoxin lipopolysaccharide (LPS) inhibits gluconeogenic gene expression and hepatic glucose output by blocking CRTC2 activation. Interleukin-1β (IL-1β) is found to disrupt gluconeogenic gene expression via the activation of the E3 ubiquitin ligase TRAF6, a key component of the Toll-like receptor 4 signaling pathway that associates with and ubiquitinates CRTC2. TRAF6 promotes the K63-linked ubiquitination of CRTC2, a modification that blocks binding of calcineurin at an adjacent calcineurin-binding site, thereby disrupting CRTC2 dephosphorylation in response to glucagon signals. Mutation of TRAF6-binding sites or ubiquitination site in CRTC2 rescues hepatic gluconeogenesis in LPS-challenged mice. These results suggest that pro-inflammatory signals intersect with the CRTC2 pathway in liver, thus contributing to hypoglycemia caused by infection.

## Introduction

Hypoglycemia is a cause of considerable morbidity and mortality in the setting of septic shock both in experimental animals and in humans [1–4]. Extensive studies have been conducted to elucidate the mechanisms of hypoglycemia under septic shock, using animal models injected with lipopolysaccharide (LPS). LPS induces hyperglycemia approximately 1 h after injection [5] followed by hypoglycemia within 6 h [6] in mice. Glycogen depletion, decreased gluconeogenesis and increased glucose consumption have been suggested as potential mechanisms of hypoglycemia induced by LPS [7–9]. However, the detailed signal pathway still remains unsolved.

Under hypoglycemia, increases in circulating glucagon promote hepatic glucose production in part through the activation of gluconeogenesis pathway by CREB coactivator CRTC2 [10]. Sequestered in the cytoplasm via phosphorylation-dependent interactions with 14-3-3 proteins, CRTC2 is dephosphorylated in response to glucagon, when it migrates to the nucleus and binds to CREB over gluconeogenic genes [11]. Glucagon stimulates CRTC2 dephosphorylation via the PKA-dependent inhibition of SIK2 and via the activation of the Ser/Thr phosphatase calcineurin [12, 13].

Here we show that in response to endotoxin (LPS) stimulation, inhibition of gluconeogenic gene expression via blocking CRTC2 activation by interleukin-1β (IL-1β) released from macrophage leads to hypoglycemia in mice. Indeed, TRAF6, the E3 ubiquitin ligase activated by IL-1β associates with and ubiquitinates CRTC2. K63-linked ubiquitination of CRTC2 by TRAF6 blocked binding of calcineurin and thereby disrupted CRTC2 dephosphorylation in response to glucagon signals. As mutation of TRAF6-binding sites in CRTC2 rescued hepatic gluconeogenesis in LPS-challenged mice, our results suggest an important regulatory node by which pro-inflammatory signals intersect with the CREB/CRTC2 pathway in liver and perhaps other tissues.

## Results

### Pro-inflammatory cytokines inhibit gluconeogenic program

**Figure 1:**
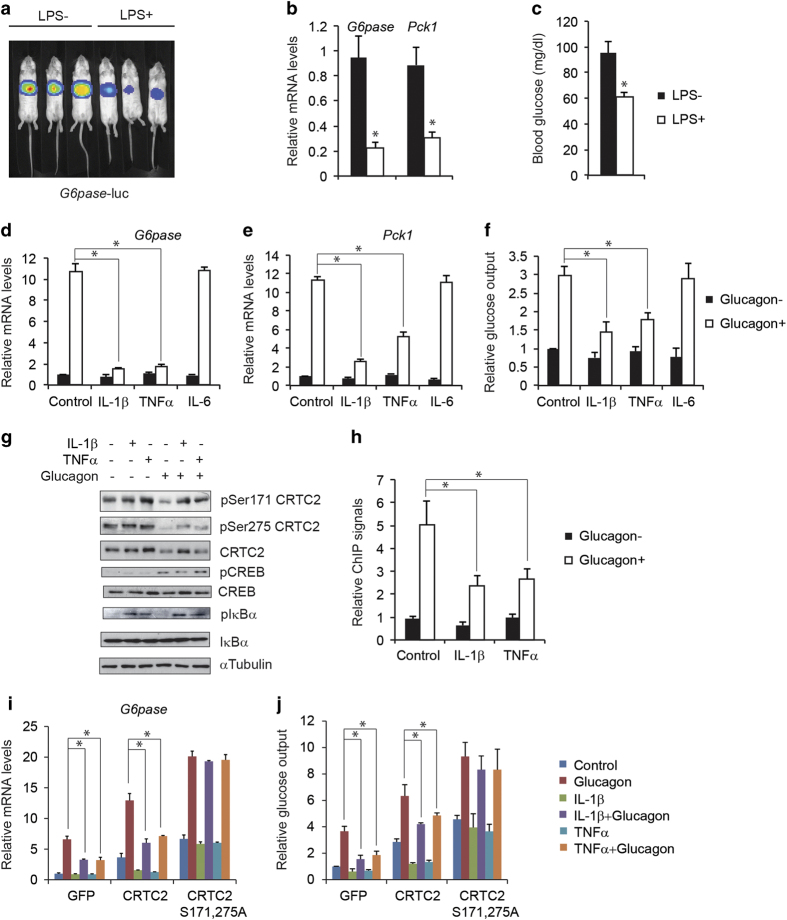
Pro-inflammatory cytokines inhibit gluconeogenic program. (**a**–**c**) Effect of LPS (30 mg kg^−1^) administration on hepatic *G6pase* luciferase reporter activity (**a**) as well as mRNA amounts for gluconeogenic genes (**b**), and circulating blood glucose concentrations (**c**) in fasted mice (*n*=9 in each group; *P<0.05). (**d**–**f**) Effect of IL-1β (10 μg l^−1^), TNFα (10 μg l^−1^) and IL-6 (10 μg l^−1^) on gluconeogenic gene expression including *G6pase* (**d**) and *Pck1* (**e**), as well as glucose output (**f**) in cultured primary hepatocytes exposed to glucagon (20 nm) (*P<0.05). (**g**) Immunoblot showing effects of IL-1β (10 μg l^−1^) and TNFα (10 μg l^−1^) on glucagon (20 nm)-induced CRTC2 dephosphorylation in primary hepatocytes. (**h**) Effect of IL-1β (10 μg l^−1^) and TNFα (10 μg l^−1^) on CRTC2 occupancy over the *G6pase* promoter in hepatocytes exposed to glucagon (20 nm) (*P<0.05). (**i**, **j**) Effect of wild type and phosphorylation-defective S171,275A CRTC2 overexpression on glucagon (20 nm)-induced *G6pase* mRNA amounts (**i**) and glucose output (**j**) in primary hepatocytes exposed to IL-1β (10 μg l^−1^) and TNFα (10 μg l^−1^) (*P<0.05).

To monitor effect of LPS on hepatic gluconeogenesis *in vivo*, we delivered *Glucose-6-phosphatase* (*G6pase)*-luc or NF-κB-luc adenovirus in mouse liver via tail-vein injection and imaged their luciferase reporter activities respectively under fasting conditions. As expected, NF-κB-luc activity increased remarkedly in the liver after LPS injection (Supplementary Figure S1a). By contrast with its stimulatory effects on the NF-κB pathway, administration of LPS acutely reduced *G6pase*-luc activity in the liver (Figure 1a). Similarly, the expression of endogenous inflammatory genes including *IL-1β* and *TNFα* was induced while the expression of gluconeogenic genes including *G6pase* and *Phosphoenolpyruvate carboxykinase 1 (Pck1)* was by contrast inhibited in the liver (Figure 1b and Supplementary Figure S1b). As a result, circulating blood glucose concentrations were dramatically decreased (Figure 1c). LPS has been proposed to modulate the gluconeogenic program indirectly via the activation of resident macrophages [14]. Supporting this scenario, exposure of primary hepatocytes to LPS had no effect on *G6pase* gene expression, whereas medium from LPS-stimulated RAW246.7 macrophages potently reduced it (Supplementary Figure S1c). Based on these results, we hypothesized that LPS-induced cytokine such as IL-1β, tumor necrosis factor-α (TNFα) or IL-6 may inhibit gluconeogensis in hepatocytes. Supporting this notion, exposure of cultured hepatocytes to IL-1β, TNFα but not IL-6 downregulated effects of glucagon on gluconeogenic gene expression (*G6pase*, *Pck1*) as well as glucose output (Figure 1d–f).

Realizing the importance of the second messenger cAMP in mediating hepatic gluconeogenesis, we tested whether pro-inflammatory cytokines disrupt gluconeogenic gene expression via the CRTC2/CREB pathway. Although exposure to IL-1β or TNFα had no effect on the PKA-mediated phosphorylation of CREB in response to glucagon (Figure 1g), IL-1β or TNFα blocked the dephosphorylation and nuclear shuttle of CRTC2 in hepatocytes exposed to glucagon (Figure 1g and Supplementary Figure S2). Glucagon-stimulated recruitment of CRTC2 to the G6pase promoter was also inhibited by IL-1β or TNFα treatment (Figure 1h). Moreover, adenoviral expression of phosphorylation-defective (S171, 275A) CRTC2 rescued G6pase gene expression and glucose output in primary hepatocytes exposed to IL-1β or TNFα, whereas wild-type CRTC2 did not (Figure 1i and j).

### Pro-inflammatory signals acutely disrupt hepatic gluconeogenesis via TRAF6-mediated inhibition of CRTC2

**Figure 2:**
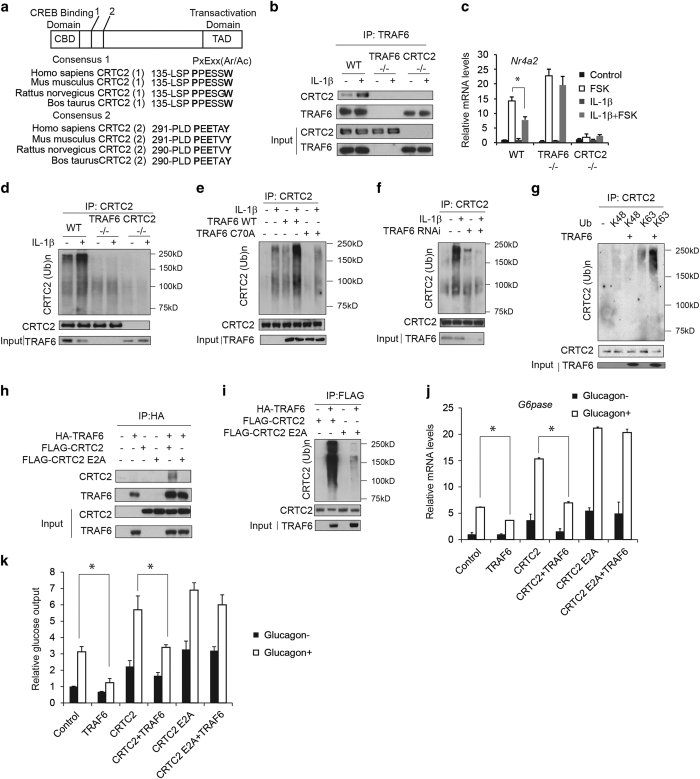
TRAF6 mediates effects of IL-1β on CRTC2 activity. (**a**) Diagram of CRTC2 and amino acid sequence alignment showing conserved TRAF6-binding motifs (PXEXXAr/Ac) in CRTC2 from different species. (**b**) Immunoblot of CRTC2 recovered from immunoprecipitates of TRAF6 prepared from wild type, TRAF6^−/−^ or CRTC2 ^−/−^ MEFs. Effect of IL-1β (10 μg l^−1^) treatment shown. (**c**) Effect of IL-1β (10 μg l^−1^) on CRTC2 target gene *Nr4a2* expression in wild type and TRAF6^−/−^ or CRTC2^−/−^ MEFs co-treated with FSK (10 μm) (*P<0.05). (**d**) Immunoblot showing amounts of ubiquitinated CRTC2 recovered from IPs of CRTC2 prepared from wild type and TRAF6^−/−^ MEFs. (**e**) Effect of TRAF6 wild type or catalytically inactive (C70A) overexpression on CRTC2 ubiquitination in primary hepatocytes exposed to IL-1β (10 μg l^−1^). (**f**) Effect of RNAi-mediated depletion of TRAF6 on CRTC2 ubiquitination in primary hepatocytes exposed to IL-1β (10 μg l^−1^). (**g**) Immunoblot showing effect of TRAF6 on amounts of ubiquitinated CRTC2 in HEK293T cells co-expressing mutant ubiquitins with mutation of all lysines except K48 or K63. (**h**) Immunoblots showing effect of mutations in the TRAF6-binding motifs (E2A) in CRTC2 on its association with TRAF6 in HEK293T cells. (**i**) Immunoblots showing effect of mutations in the TRAF6-binding motifs (E2A) in CRTC2 on its ubiquitination in HEK293T cells. (**j**, **k**) Effect of TRAF6 overexpression on wild type and E2A CRTC2 induced *G6pase* mRNA amounts (**j**) and glucose output (**k**) in primary hepatocytes exposed to glucagon (20 nm) (*P<0.05).

TRAF6, a key component of IL-1β signal pathway, has been found to associate with relevant substrates containing a conserved PXEXXAr/Ac (Ar/Ac for an aromatic or acidic residue) motif [15]. We found that CRTC2 contains two well-conserved consensus PXEXXAr/Ac motifs, suggesting its interaction with TRAF6 (Figure 2a). We confirmed the CRTC2:TRAF6 interaction in wild type but not TRAF6^−/−^ or CRTC2^−/−^ MEFs, under basal conditions and to a greater extent following exposure to IL-1β, by co-immunoprecipitation assay of endogenous proteins (Figure 2b). Supporting a functional role for TRAF6, exposure to IL-1β decreased CRTC2/CREB target gene *Nuclear receptor subfamily 4, group A, member 2 (Nr4a2)* expression in wild-type mouse embryonic fibroblasts (MEFs), but it had only modest effects in TRAF6^−/−^ MEFs or CRTC2^−/−^ MEFs co-stimulated with Forskolin (FSK) (Figure 2c). Restoring TRAF6 expression in TRAF6^−/−^ MEFs or CRTC2 expression in CRTC2^−/−^ MEFs rescued the effect (Supplementary Figure S3a and b). Furthermore, overexpression of wild-type TRAF6 but not catalytically inactive (C70A) TRAF6 reduced gluconeogenic gene (*G6pase*, *Pck1*) expression and glucose output as well as CRTC2 occupancy over the *G6pase* promoter in hepatocytes exposed to glucagon (Supplementary Figure S4a–d), while RNAi-mediated depletion of TRAF6 increased it (Supplementary Figure S4e–h). Taken together, these results indicate that IL-1β inhibits CRTC2 signaling through TRAF6.

We considered that TRAF6 may promote CRTC2 ubiquitination. Exposure to IL-1β increased CRTC2 ubiquitination in wild type but not TRAF6^−/−^ or CRTC2^−/−^ MEFs (Figure 2d). Similarly, overexpression of wild type but not TRAF6 C70A potentiated effects of IL-1β on CRTC2 ubiquitination (Figure 2e), whereas RNAi-mediated depletion of TRAF6 reduced amounts of ubiquitinated CRTC2 (Figure 2f) in primary hepatocytes.

E3 ubiquitin ligases have been shown to regulate substrate degradation or cellular trafficking via K48 or K63-linked ubiquitination, respectively [16–18]. Consistent with its effects on other cellular proteins, TRAF6 appeared to ubiquitinate CRTC2 selectively via K63-dependent linkages; overexpression of K63-only ubiquitin promoted TRAF6-dependent ubiquitination of CRTC2 while K48-only mutant ubiquitin did not (Figure 2g).

Within the CRTC family, CRTC1 and CRTC2 contain well-conserved consensus PXEXXAr/Ac motifs, whereas CRTC3 does not (Supplementary Figure S5a). Correspondingly, TRAF6 was found to associate with and promote the ubiquitination of both CRTC1 and CRTC2 but not CRTC3 (Supplementary Figure S5b). As a result, overexpression of TRAF6 reduced *G6pase* reporter activity induced by both CRTC1 and CRTC2 but not CRTC3 (Supplementary Figure S5c). Moreover, mutation of the putative interaction motifs in CRTC1 and CRTC2 disrupted TRAF6 binding and ubiquitination (Figure 2h and i and Supplementary Figure S5d and e). Consistently, TRAF6 reduced *G6pase* gene expression or reporter activity induced by wild-type CRTC1 and CRTC2 but not TRAF6-interaction-defective CRTC1 and CRTC2 (Figure 2j and Supplementary Figure S5f). As a result, glucose output induced by wild-type CRTC2 but not TRAF6-interaction-defective CRTC2 was inhibited by TRAF6 (Figure 2k).

TRAF2, a counterpart of TRAF6 in TNFα signal pathway, also interacted with CRTC2 and promoted ubiquitination of CRTC2 (Supplementary Figure S6a and b). Similarly, overexpression of wild-type TRAF2 but not catalytically inactive (C34A) TRAF2 reduced *G6pase* reporter activity (Supplementary Figure S6c).

**Figure 3:**
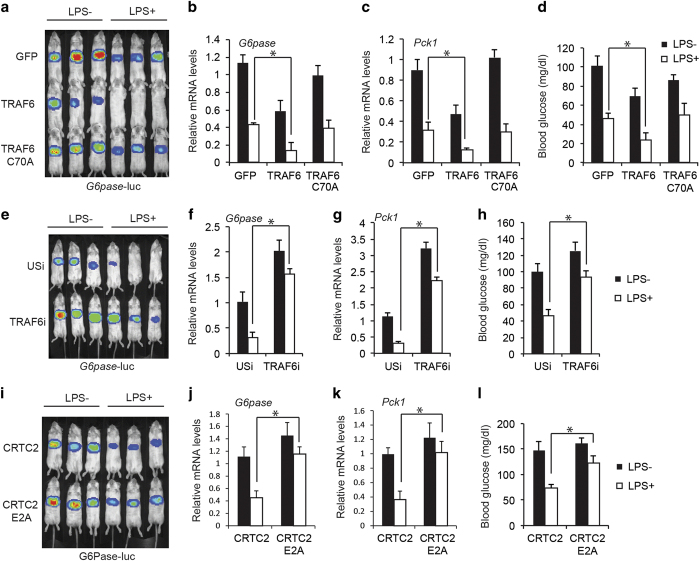
Pro-inflammatory signals acutely disrupt hepatic gluconeogenesis via TRAF6-mediated inhibition of CRTC2. (**a**–**d**) Effect of adenoviral TRAF6 wild type or catalytically inactive C70A overexpression on hepatic *G6pase* reporter activity (**a**), as well as mRNA amounts for gluconeogenic genes including *G6pase* (**b**) and *Pck1* (**c**) and circulating blood glucose concentrations (**d**) in fasted mice following administration of LPS (30 mg  kg^−1^) (*n*=9 in each group; *P<0.05). (**e**–**h**) Effect of adenoviral RNAi-mediated knockdown of TRAF6 on hepatic *G6pase* reporter activity (**e**), as well as mRNA amounts for gluconeogenic genes including *G6pase* (**f**) and *Pck1* (**g**), and circulating blood glucose concentrations (**h**) in fasted mice following administration of LPS (30 mg kg^−1^) (*n*=9 in each group; *P<0.05). (**i**–**l**) Effect of wild type and TRAF6 interaction-defective CRTC2 (E2A) on hepatic *G6pase* reporter activity (**i**) as well as mRNA amounts for gluconeogenic genes including *G6pase* (**j**) and *Pck1* (**k**), and circulating blood glucose concentrations (**l**) in fasted mice following administration of LPS (30 mg kg^−1^) (*n*=9 in each group; *P<0.05).

Based on these results, we evaluated whether TRAF6 mediates inhibitory effects of LPS on the gluconeogenic program *in vivo*. LPS administration (i.p.) decreased *G6pase*-luc activity as well as gluconeogenic gene expression (*G6pase* and *Pck1*) and circulating blood glucose concentrations in fasted mice; these effects were potentiated by overexpression of wild type but not catalytically inactive C70A mutant TRAF6 (Figure 3a–d). Conversely, RNAi-mediated knockdown of TRAF6 enhanced *G6pase*-luc activity as well as gluconeogenic gene expression (*G6pase* and *Pck1*) and circulating glucose concentrations in fasted mice, indicating that TRAF6 modulates hepatic gluconeogenesis in part through inhibition of CRTC2 (Figure 3e–h). Pointing to an important role for the TRAF6:CRTC2 interaction, effects of LPS on the gluconeogenic profile were diminished in mice expressing TRAF6-interaction-defective CRTC2 relative to wild-type CRTC2 (Figure 3i–l). Collectively, these results suggest that TRAF6 mediates inhibitory effects of a pro-inflammatory signal on CRTC2 activity in liver.

### TRAF6-mediated ubiquitination of CRTC2 disrupts its dephosphorylation via CnA

**Figure 4:**
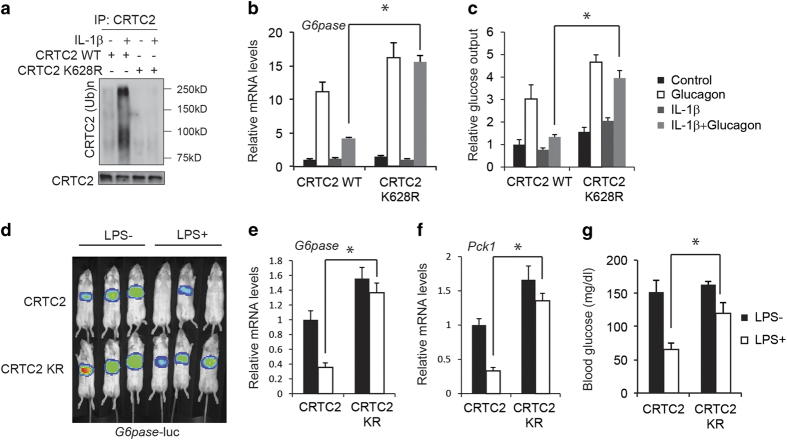
TRAF6-mediated ubiquitination of CRTC2 inhibits its activity. (**a**) Immunoblot showing ubiquitination of wild type and K628R mutant CRTC2 in hepatocytes exposed to IL-1β (10 μg l^−1^). (**b**, **c**) Effects of wild type or ubiquitination-defective K628R CRTC2 on *G6pase* mRNA amounts (**b**) and glucose output (**c**) in hepatocytes exposed to IL-1β (10 μg l^−1^) and glucagon (20 nm) (*P<0.05). (**d**–**g**) Effect of wild type or K628R mutant CRTC2 on hepatic *G6pase* reporter activity (**d**) as well as mRNA amounts for gluconeogenic genes including *G6pase* (**e**) and *Pck1* (**f**), and circulating blood glucose concentrations (**g**) in fasted mice following administration of LPS (30 mg k^−1^g) (*n*=9 in each group; *P<0.05).

We examined the mechanism by which TRAF6-mediated ubiquitination inhibits CRTC2 activity. Based on mass spectrometry studies showing that CRTC2 undergoes ubiquitination at Lys628 [11], we tested whether this site is ubiquitinated by TRAF6. Supporting this idea, exposure to IL-1β increased CRTC2 ubiquitination in hepatocytes expressing wild-type CRTC2, but it had no effect in cells expressing K628R mutant CRTC2 (Figure 4a). Correspondingly, exposure to IL-1β reduced glucagon-stimulated *G6pase* gene expression in cells expressing wild-type CRTC2, but not in K628R CRTC2-expressing cells (Figure 4b). Furthermore, while exposure to IL-1β had only modest effects on CRTC2/CREB target gene *Nr4a2* expression in CRTC2^−/−^ MEFs co-stimulated with FSK, overexpression of wild-type CRTC2 but not CRTC2 E2A or CRTC2 KR in CRTC2^−/−^ MEFs rescued the effect (Supplementary Figure S3a). As a result, K628R mutant CRTC2 rescued hepatic *G6pase* reporter activity as well as gluconeogenic gene expression (*G6pase* and *Pck1*) and circulating blood glucose concentrations in mice administered LPS, whereas wild-type CRTC2 did not (Figure 4c–g). It has been reported that constitutive photomorphogenic protein 1 (COP1) interacts with CRTC2 and promotes the K48 ubiquitination and degradation of CRTC2 at Lys628 [12]. However, COP1 interaction-defective CRTC2 mutant (CRTC2 V214, P215A, CRTC2 VP/AA) was still able to be ubiquitinated by TRAF6 and exposure to IL-1β reduced glucagon-stimulated *G6pase* gene expression in cells expressing either wild-type CRTC2 or VP/AA CRTC2 (Supplementary Figure S7a and b). Furthermore, IL-1β induced CRTC2 ubiquitination at a similar level in either USi-infected or COP1i-infected cells (Supplementary Figure S7c). Consistently, when primary hepatocytes were infected with adenovirus encoding either unspecific RNAi (USi) or COP1 RNAi (COP1i), exposure to IL-1β reduced glucagon-stimulated *G6pase* gene expression in cells expressing either USi or COP1i (Supplementary Figure S7d), excluding COP1 participation in this setting. Taken together, these results indicate that pro-inflammatory cytokines inhibit the CRTC2 pathway via TRAF6-dependent ubiquitination of CRTC2 at Lys628.

**Figure 5:**
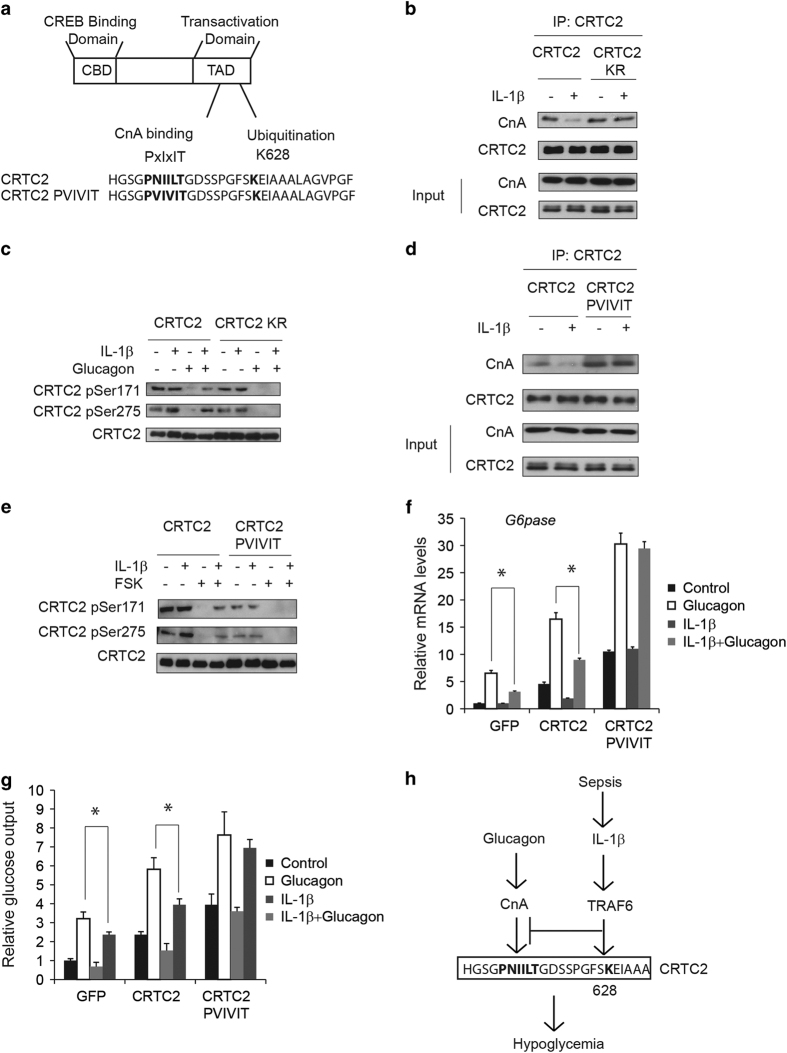
TRAF6-mediated ubiquitination of CRTC2 disrupts its dephosphorylation via CnA. (**a**) Diagram and sequence of CRTC2 CnA-binding sites and ubiquitination site. (**b**) Effect of IL-1β (10 μg l^−1^) on CnA binding of wild type or K628R mutant CRTC2. (**c**) Effect of IL-1β (10 μg l^−1^) on dephosphorylation of wild type or K628R mutant CRTC2 in primary hepatocytes exposed to glucagon (20 nm). (**d**) Effect of IL-1β (10 μg l^−1^) on CnA binding of wild-type CRTC2 or mutant CRTC2 containing a high affinity CnA-binding site (PVIVIT). (**e**) Effect of IL-1β (10 μg l^−1^) on dephosphorylation of wild type or PVIVIT CRTC2 in primary hepatocytes exposed to glucagon (20 nm). (**f**, **g**) Effect of wild type and PVIVIT CRTC2 on *G6pase* mRNA amounts (**f**) and glucose output (**g**) in hepatocytes exposed to IL-1β (10 μg l^−1^) and glucagon (20 nm) (*P<0.05). (**h**) Schematic of proposed mechanism. In response to septic shock, pro-inflammatory cytokines such as IL-1β stimulates CRTC2 ubiquitination at Lys628 through TRAF6, which in turn blocks CRTC2 activation by disrupting CnA binding.

Glucagon has been shown to promote CRTC2 dephosphorylation via induction of calcineurin (CnA) [19]. Interestingly, Lys628 is located adjacent to a calcineurin-binding site (613-PNIILT-618) in CRTC2 (Figure 5a), leading us to consider that TRAF6-dependent ubiquitination might interfere with CnA binding and dephosphorylation of CRTC2. In co-immunoprecipitation studies, exposure to IL-1β decreased the association of CRTC2 with CnA and correspondingly blocked the dephosphorylation of CRTC2 at the Ser171 and Ser275 sites (Figure 5b and c). In contrast with wild-type CRTC2, IL-1β had no effect on CnA binding or dephosphorylation of ubiquitination-defective K628R CRTC2 (Figure 5b and c). Similarly, substitution of the native CnA-binding site (PNIILT) in CRTC2 with an optimized high affinity site (PVIVIT) [20, 21] increased CnA binding and CRTC2 dephosphorylation even in the presence of IL-1β co-stimulation (Figure 5d and e). As a result, exposure to IL-1β reduced *G6pase* gene expression as well as glucose output induced by wild-type CRTC2 but not PVIVIT mutant CRTC2 in primary hepatocytes (Figure 5f and g). Collectively, these results demonstrate that pro-inflammatory stimuli disrupt signaling through the CRTC2 pathway in part via the TRAF6-mediated ubiquitination and inhibition of CRTC2 dephosphorylation.

## Discussion

During hypoglycemia, increases in circulating glucagon trigger CRTC2 dephosphorylation in part by increasing its association with CnA at a consensus PXIXIT motif [19]. Here we show that in response to pro-inflammatory cytokines, TRAF6 stimulates CRTC2 ubiquitination at Lys628, which in turn blocks CRTC2 activation by disrupting CnA binding (Figure 5h). Most importantly, RNAi-mediated knockdown of TRAF6 or expression of TRAF6-interaction-defective CRTC2 and TRAF6-ubiquination-defective CRTC2 in liver rescues the hypoglycemic phenotype induced by LPS injection. Taken together, our results unveil a novel mechanism by which pro-inflammatory signals intersect with the CRTC2 pathway in liver and this mechanism contributes to the hypoglycemia caused by infection.

Post-transcriptional modification at Lys628 of CRTC2 plays important roles in regulating CRTC2 functions. COP1 mediates CRTC2 K48 ubiquitination at Lys628 and promotes CRTCs degradation under feeding signal such as insulin stimulation, which serves as a mechanism for insulin-mediated suppression of gluconeogenesis [12]. On the contrary, fasting signal such as glucagon stimulation activates p300/CBP and p300/CBP in turn acetylates CRTC2 at Lys628, which protects CRTC2 against ubiquitin-mediated degradation [11]. Collectively, these mechanisms help to maintain normal glucose levels via counter-regulatory effects of insulin and glucagon on hepatic gluconeogenesis and dysfunction of these mechanisms leads to hyperglycemia in diabetes. Here, our work contributes to the importance of Lys628 modification on CRTC2 functions by showing that inflammation signal stimulates K63 ubiquitination of Lys628 through TRAF6. Although K63 ubiquitination of CRTC2 does not lead to protein degradation, it blocks the binding of CnA to CRTC2 and dephosphorylation of CRTC2. Further investigation on Lys628 methylation or sumoylation will unveal more physiological stimulus that could regulate gluconeogensis through CRTC2.

It has been reported that endotoxin-induced hypoglycemia is paralleled by an increase in counteregulatory hormones such as glucagon as well as gluconeogenic precursor supply (lactate and gluconeogenic amino acids) [22, 23]. However, hepatic gluconeogenesis is severely impaired under the same settings, representing a ‘glucagon resistance’ phenotype [24–26]. In the present study, we show that the glucagon-stimulated signal pathway is defective due to the impaired dephosphorylation of CRTC2 by TRAF6 ubiquitination, a situation quite similar to the impaired insulin signal transduction under insulin resistance status. Thus, acute septic shock causes hypoglycemia via induction of glucagon resistance, while chronic low-grade inflammation leads to hyperglycemia via induction of insulin resistance. The detailed mechanism differences between these two pathological conditions still remain unclear.

Glucocorticoid receptor signaling plays an important role in the regulation of gluconeogenesis [27] and is inhibited by septic shock, although the mechanism has not been clarified [6, 28]. CRTC2 functions as a coactivator for the glucocorticoid receptor and is required for the glucocorticoid-associated induction of hepatic gluconeogenesis [29]. Besides, glucose uptake in periphery tissue including skeletal muscle and adipose tissue helps to maintain euglycemia under physiological conditions. Glucose uptake is increased following LPS challenge, which contributes to the hypoglycemia caused by infection [30–32]. CRTC2 is reported to inhibit glucose transporter type 4 (Glut4) expression and glucose uptake through ATF3 [33]. Thus, TRAF6-mediated inhibition of CRTC2 may serve as a broad mechanism of septic shock-induced hypoglycemia in multiple tissues, which will need further investigation.

## Materials and Methods

### Cells, antibodies and reagents

Primary hepatocytes were prepared as described [34]. HEK293T and RAW 264.7 (mouse leukemic monocyte macrophage cell line) cells were obtained from the American Type Culture Collection (ATCC, Manassas, VA, USA) and maintained in Dulbecco's modified Eagle's medium with 10% fetal bovine serum. RAW 264.7 cells were stimulated with LPS for 16 h. Fetal bovine serum-free medium supernatants were collected and supplied to mouse primary hepatocytes. MEFs from wild type and CRTC2^−/−^ mice were prepared as previously described [35]. MEFs from wild type and TRAF6^−/−^ mice were kindly provided by Dr Hui-Kuan Lin (The University of Texas MD Anderson Cancer Center, Houston, TX, USA). All cells were recently authenticated and tested for contamination. Anti-pSer171 CRTC2 (1:1 000), pSer275 CRTC2 (1:1 000), CRTC2 (1:5 000) antibodies were kindly provided by Dr Marc Montminy (Salk Institute, La jolla, CA, USA) [12, 36]. Anti-pSer 32/36 IκBα (1:1 000; cat. no. 2859), IκBα (1:1 000; cat. no. 4812), TRAF6 (1:1 000; cat. no. 8028), Ubiquitin (1:1 000; cat. no. 3936) and CnA (1:1 000; cat. no. 2614) antibodies were purchased from Cell signaling Technology (Danvers, MA, USA). Anti-α-tubulin (1:5 000; cat. no. ab18251) antibody was purchased from Abcam (Cambridge, UK).

### Animals and adenovirus

Eight- to 10-week-old and weight-matched male C57BL/6J mice were purchased from Shanghai Laboratory Animal Center (Shanghai, China) and were adapted to colony cages with 12 h light/dark cycle in a temperature-controlled environment with free access to water and standard irradiated rodent diet (5% fat; Research Diet, D12450, New Brunswick, NJ, USA). For adenovirus injection, 10^9^ plaque-forming units (p.f.u.) Ad-*G6pase*-luc or Ad-NF-κB-luc and 5×10^7^ p.f.u. Ad-RSV-β-gal, 1×10^8^ p.f.u. Ad-GFP, Ad-TRAF6, Ad-unspecific RNAi (USi), Ad-TRAF6 RNAi (TRAF6i), Ad-CRTC2, Ad-CRTC2 E2A and Ad-CRTC2 K628R were delivered by tail-vein injection. TRAF6 RNAi was constructed using the sequence 5′- GGGCGAGCTCAAACGGACCATT-3′. All animal studies were approved by the Animal Experiment Committee of Tongji University and in accordance with the guidelines of School of medicine, Tongji University.

### *In vivo* imaging

Mice were imaged on days 3–5 after adenovirus delivery. Mice were fasted for 12 h starting at Zeitgeber time (ZT) 12 and injected intraperitoneally with LPS (30 mg kg^−1^) for 1 h. Before imaging, mice were injected intraperitoneally with 50 mg kg^−1^ Nembutal (Abbott Laboratories, Lake Bluff, IL, USA) and 100 mg kg^−1^ sterile firefly D-luciferin (Qianchen, Shanghai, China). Mice were imaged using the IVIS 100 Imaging System (PerkinElmer, Waltham, MA, USA) and image data were analyzed using Living Image software (Xenogen, Hudson, NY, USA) as described [11]. Liver samples were taken immediately after imaging and lysates were subjected to β-galactosidase assay to ensure the equal level of adenoviral infection.

### *In vitro* analysis

Mouse tissues were sonicated at 4 °C, centrifuged and supernatants were reserved for β-gal activity, protein determinations, SDS-PAGE analysis and quantitative analysis. Blood glucose levels were measured with a One Touch Ultra Glucometer (Johnson & Johnson, New Brunswick, NJ, USA).

### Glucose output

Glucose output from primary hepatocytes was determined enzymatically. Cells were pre-treated with IL-1β (10 μg l^−1^), TNFα (10 μg l^−1^) and IL-6 (10 μg l^−1^) for 15 min and then stimulated by glucagon (20 nm) or FSK (10 μm) for 1 h in glucose-free M199 media supplemented with 10 mm lactate and 1 mm pyruvate. Glucose levels in the medium was measure by Glucose (GO) Assay Kit from Sigma-Aldrich (St Louis, MO, USA)

### Quantitative real-time-PCR and immunoblot

Cells were pre-treated with IL-1β (10 μg l^−1^), TNFα (10 μg l^−1^) and IL-6 (10 μg l^−1^) for 15 min and then stimulated by glucagon (20 nm) or FSK (10 μm) for 2 h. Total RNA was isolated by using TRIzol reagent (Invitrogen, Carlsbad, CA, USA) and reverse transcription was done using FastQuant RT kit (Tiangen, Shanghai, China). Real-time PCR was carried out using SuperReal SYBR Green kit (Tiangen Shanghai, China) and Lightcycler 96 (Roche, Penzberg, Germany). β-Actin was used as a reference gene. Cells were pre-treated with IL-1β (10 μg l^−1^), TNFα (10 μg l^−1^) and IL-6 (10 μg l^−1^) for 15 min and then stimulated by glucagon (20 nm) or FSK (10 μm) for 30 min. Immunoblot and immunoprecipitation were performed as described [37].

### CRTC2 ubiquitination

*In vivo* ubiquitination assays were performed as described [12]. In brief, cells treated with IL-1β (10 μg l^−1^) for 30 min were lysed by lysis buffer containing 100 mm Tris-HCl, pH 8.0, and 6 M urea. Lysates were diluted and anti-CRTC2 immunoprecipitates were prepared. Ubiquitinated CRTC2 was detected by immunoblot.

### Chromatin immunoprecipitation

Cultured mouse primary hepatocytes were plated in 150 mm plates and pre-treated with IL-1β (10 μg l^−1^) or TNFα (10 μg l^−1^) for 30 min, then exposed to glucagon for 1 h. Chromatin immunoprecipitation assays were performed as described previously, with minimal modification [37]. In brief, the treated hepatocytes were crosslinked with 1% formaldehyde for 15 min. Crosslinking reactions were stopped with 0.125 m glycine. Crosslinked cells were washed in phosphate-buffered saline three times and stored at −80 °C before use. Fragmented, precleared chromatin lysate was incubated overnight with indicated antibodies. The primers for *G6pase* promoter (5′- GGAGGGCAGCCTCTAGCACTGTCAA-3′, 5′- TCAGTCTGTAGGTCAATCCAGCCCT-3′) were used for chromatin immunoprecipitation analysis as described [38]. All signals were normalized to the input chromatin signals.

### Statistical analysis

All studies were performed on at least three independent occasions. Results are reported as mean±s.e.m. For all viability experiments, a two-tailed Student’s *t*-test or two-way Anova test was used to evaluate statistical significance, which was noted when *P<0.05 or **P<0.01.
