# Supplementary material for: Suppression of CRTC2-mediated hepatic gluconeogenesis by TRAF6 contributes to hypoglycemia in septic shock
Source: Cell Discov. 2016 Dec 13;2:16046–. doi: 10.1038/celldisc.2016.46 (PMC5153457; doi:10.1038/celldisc.2016.46)
Supplement: Supplementary Information [file celldisc201646-s1.pdf]

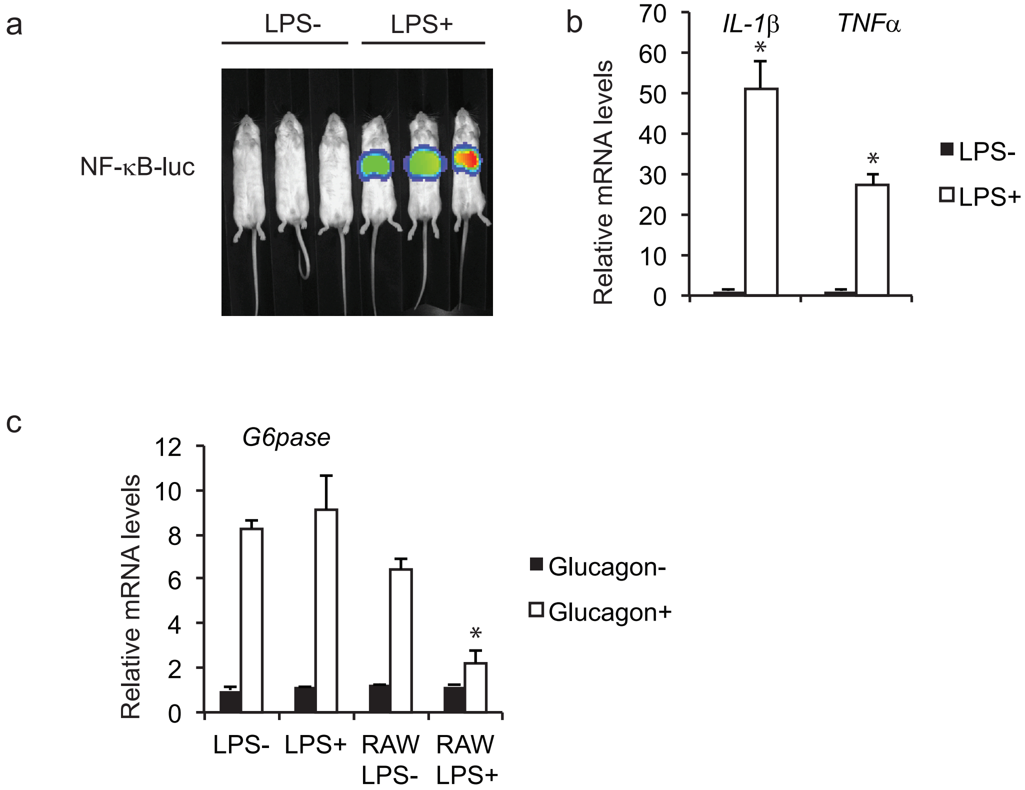

Supplementary Figure S1: Pro-inflammatory cytokines stimulate NF-κB activation in liver. (a-b) Effect of LPS (30 mg/kg) administration on hepatic NF-κB luciferase reporter activity (a) as well as mRNA amounts for pro-inflammatory genes including *IL-1β* and *TNFα* (b) in fasted mice. ($n = 9$ in each group; *$p < 0.05$). (c) Effect of LPS (1 mg/l) or medium from LPS (1 mg/l)-stimulated RAW macrophages on *G6pase* mRNA amounts in primary hepatocytes exposed to glucagon. (*$p < 0.05$).

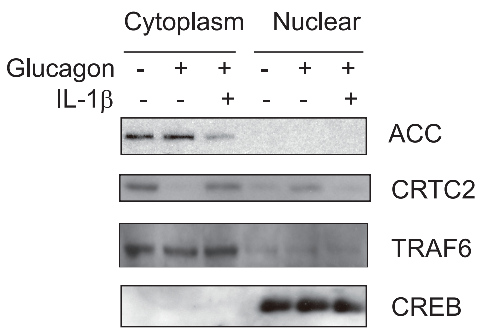

Supplementary Figure S2: IL-1β inhibits CRTC2 nuclear translocation in primary hepatocytes.
Immunoblot showing effects of IL-1β (10 μg/l) on glucagon (20nM)-induced CRTC2 nuclear translocation in primary hepatocytes. Acetyl-CoA carboxylase (ACC) was used as cytosol marker. CREB was used as nuclear marker.

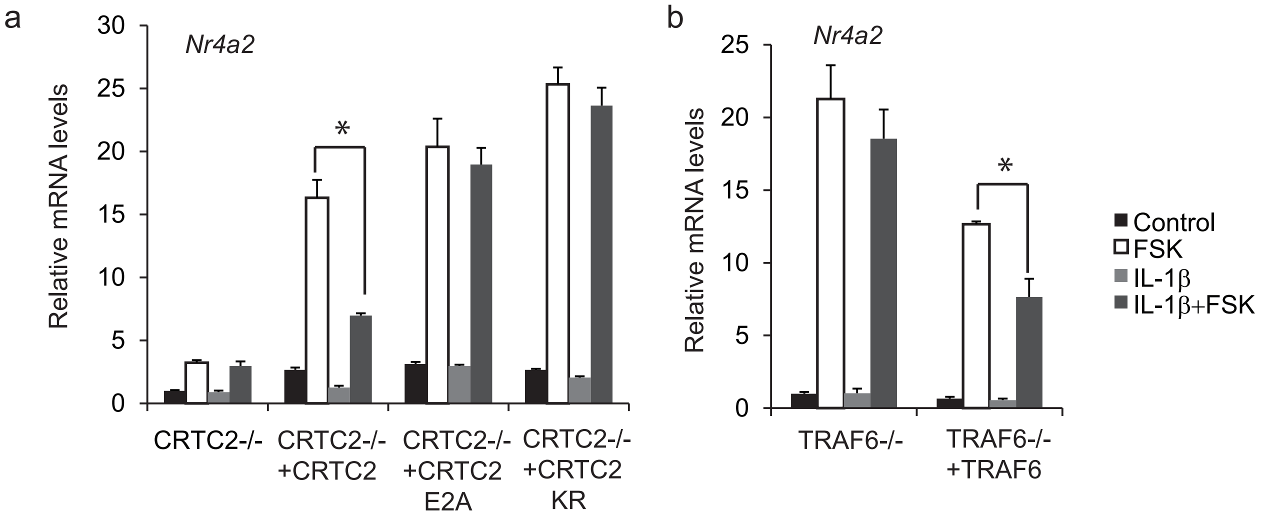

Supplementary Figure S3: TRAF6 inhibits CRTC2 activity in MEFs.
(a) Effect of IL-1β (10 μg/l) on FSK (10 μM)-stimulated *Nr4a2* expression in CRTC2-/- MEFs transfected with wild-type, E2A CRTC2 or K628R CRTC2. (*$p < 0.05$). (b) Effect of IL-1β (10 μg/l) on FSK (10 μM)-stimulated *Nr4a2* expression in TRAF6-/- MEFs transfected with or without TRAF6. (*$p < 0.05$).

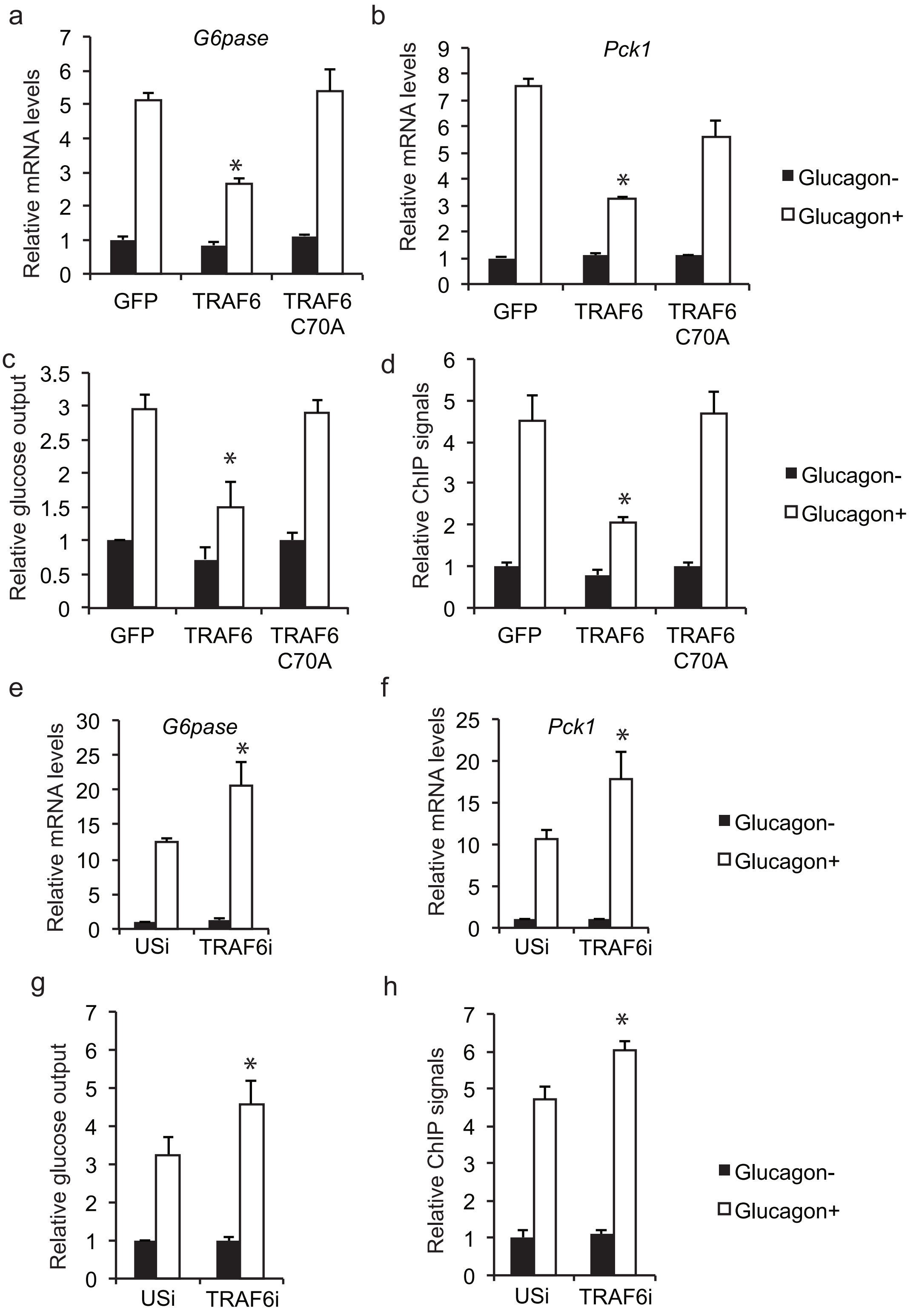

Supplementary Figure S4: TRAF6 inhibits CRTC2 activity in primary hepatocytes. (a-d) Effect of TRAF6 wild-type or catalytically inactive (C70A) over-expression on gluconeogenic gene expression including *G6pase* (a) and *Pck1* (b), as well as glucose output (c) and CRTC2 occupancy over the *G6pase* promoter (d) in cultured primary hepatocytes exposed to glucagon (20nM). (*p < 0.05). (e-h) Effect of RNAi-mediated depletion of TRAF6 on gluconeogenic gene expression including *G6pase* (e) and *Pck1* (f), as well as glucose output (g) and CRTC2 occupancy over the *G6pase* promoter (h) in cultured primary hepatocytes exposed to glucagon (20nM). (*p < 0.05).

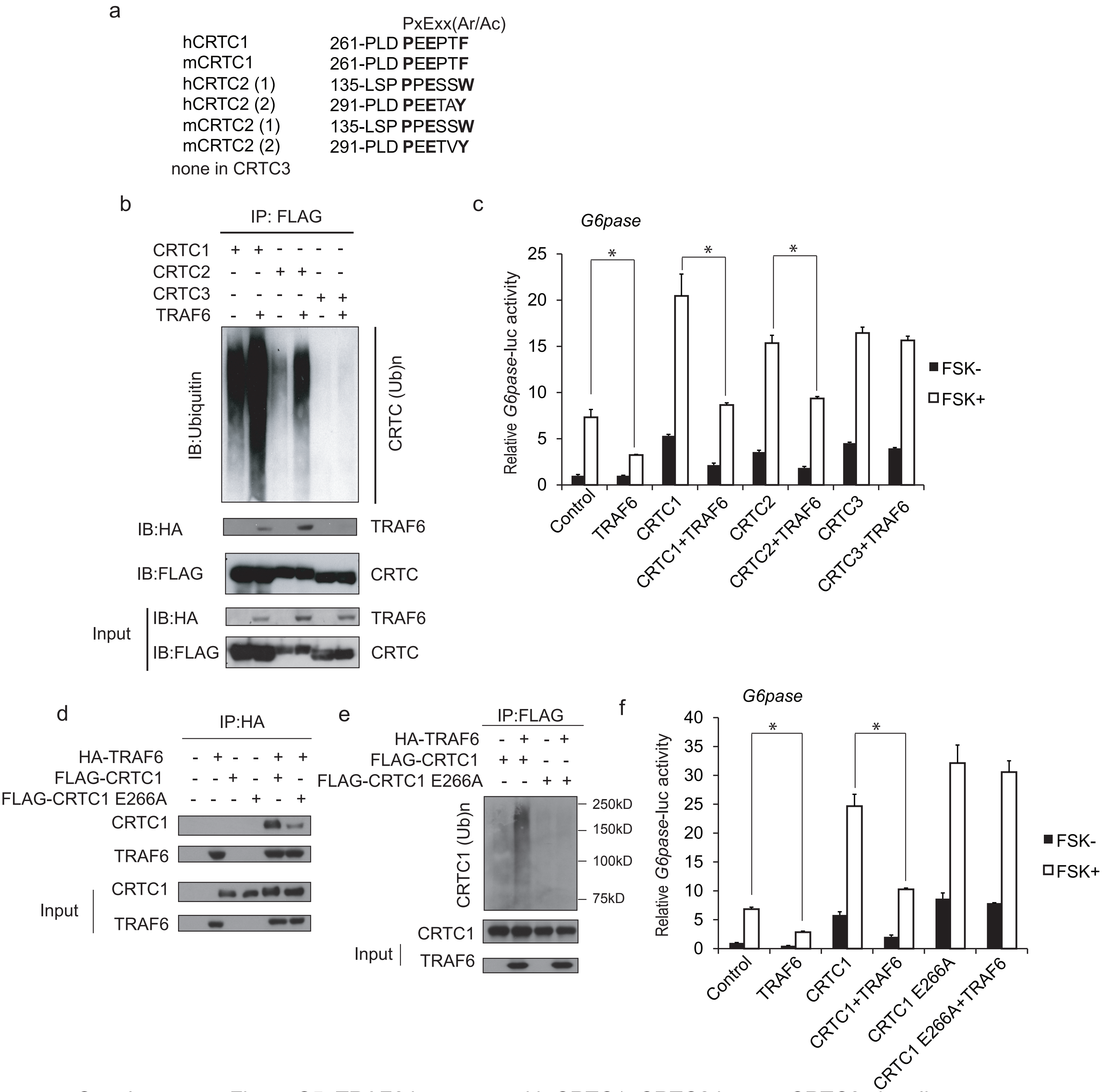

Supplementary Figure S5: TRAF6 intersects with CRTC1, CRTC2 but not CRTC3 signaling. (a) Sequence alignment showing conserved TRAF6 binding motifs (PXEXXAr/Ac) in mouse CRTC family members. (b) Immunoblot showing amounts of CRTC family members recovered from immunoprecipitates of TRAF6 and amounts of ubiquitinated-CRTC family members recovered from IPs of CRTC family members prepared from HEK293T cells. (c) Effect of TRAF6 overexpression on CRTC family members induced *G6pase* reporter activity in HEK293T cells exposed to FSK (10 μM). (*$p < 0.05$). (d) Immunoblots showing effect of mutations in the TRAF6 binding motifs (E266A) in CRTC1 on its association with TRAF6 in HEK293T cells. (e) Immunoblots showing effect of mutations in the TRAF6 binding motifs (E266A) in CRTC1 on its ubiquitination in HEK293T cells. (f) Effect of TRAF6 overexpression on wild-type and E266A CRTC1 induced *G6pase* reporter activity in HEK293T cells exposed to FSK (10 μM). (*$p < 0.05$).

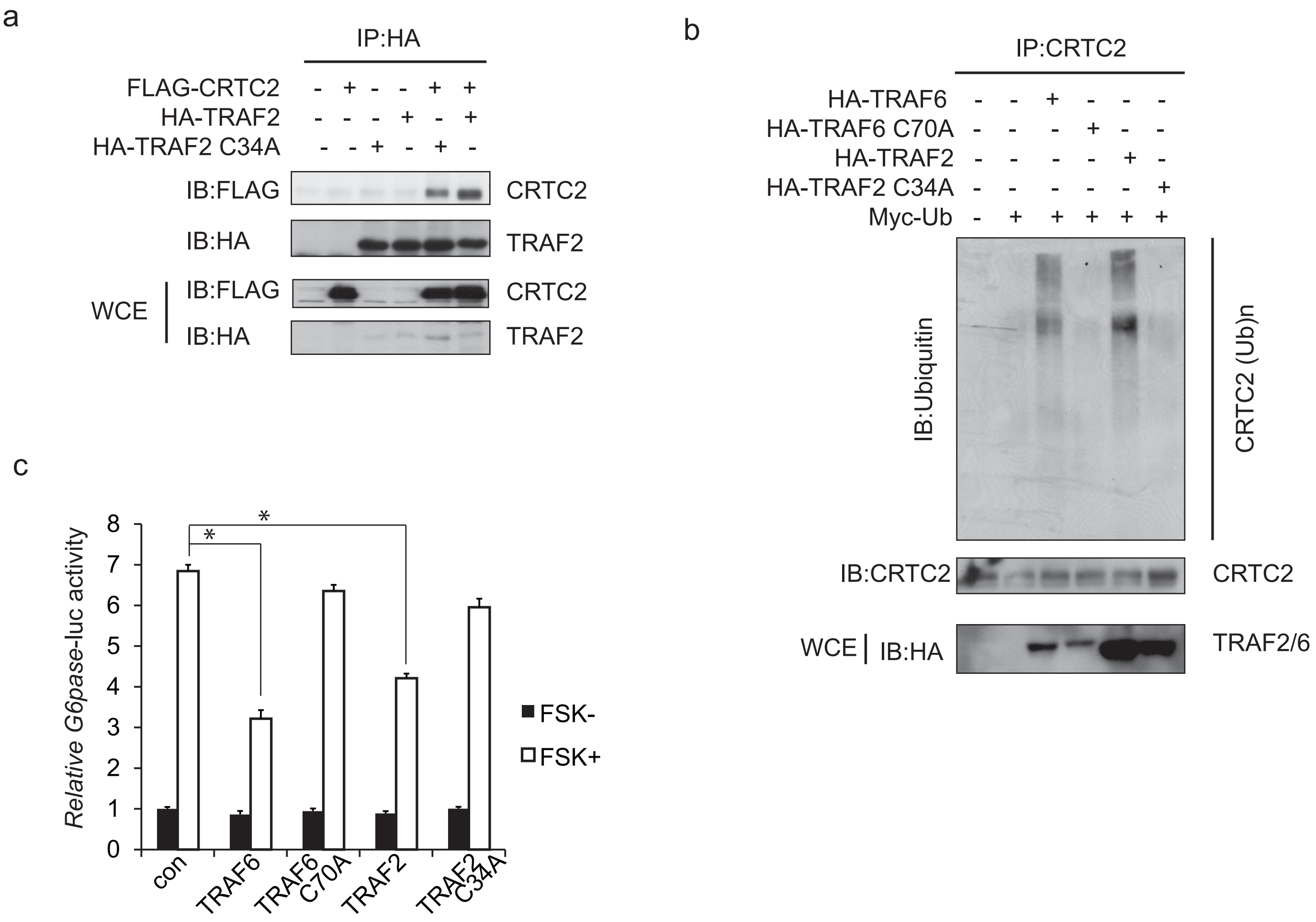

Supplementary Figure S6: TRAF2-mediated ubiquitination of CRTC2 inhibits its activity. (a) Immunoblot of CRTC2 recovered from immunoprecipitates of TRAF2 prepared from HEK293T cells. (b) Effect of TRAF6 wild-type or catalytically inactive (C70A) and TRAF2 wild-type or catalytically inactive (C34A) over-expression on CRTC2 ubiquitination in HEK293T cells. (c) Effect of TRAF6 wild-type or catalytically inactive (C70A) and TRAF2 wild-type or catalytically inactive (C34A) over-expression on *G6pase* reporter activity in HEK293T cells exposed to FSK (10 μM). (*$p < 0.05$).

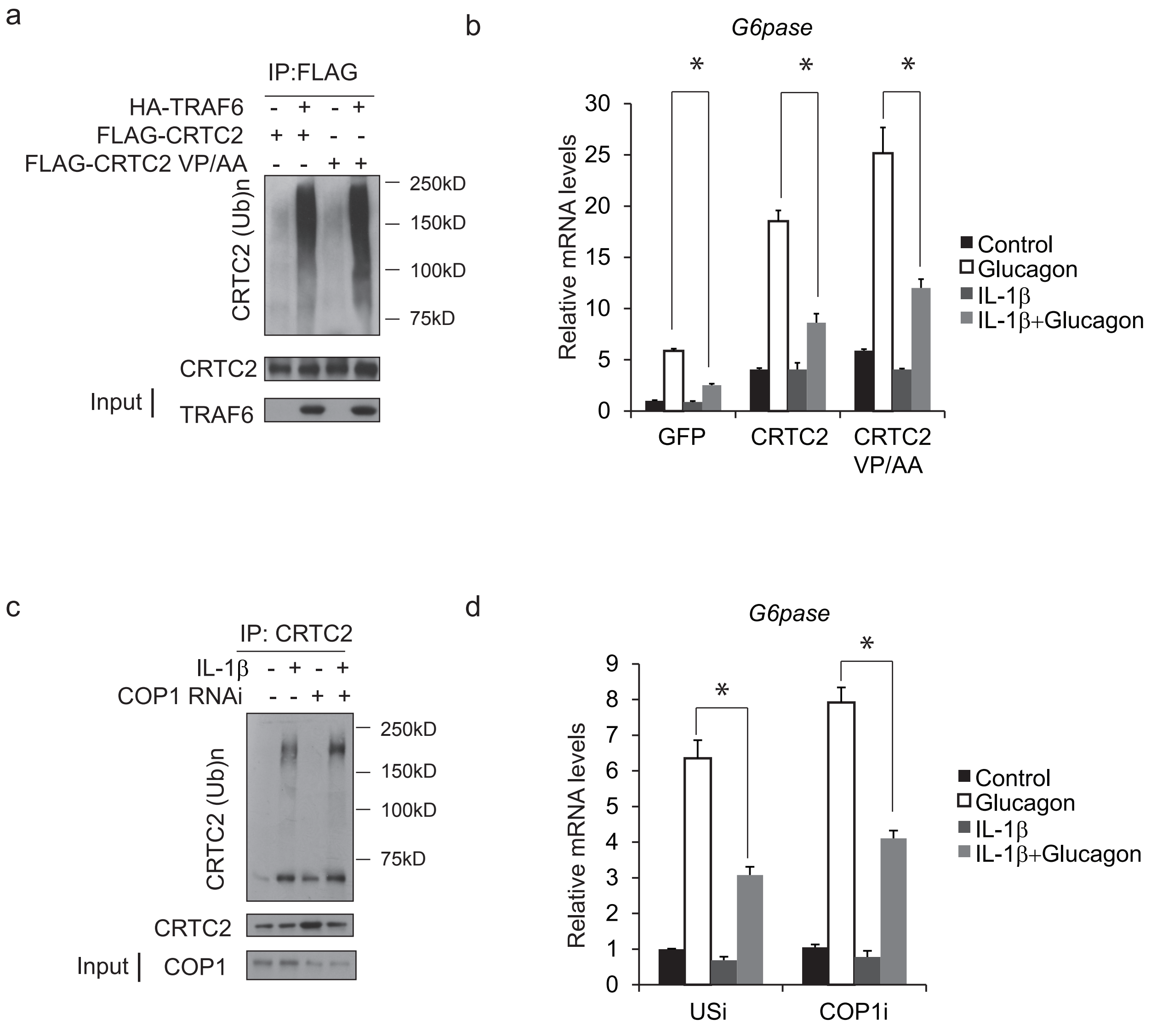

Supplementary Figure S7: COP1-mediated ubiquitination of CRTC2 does not mediate effects of IL-1β on CRTC2 activity.
(a) Immunoblots showing effect of mutations in the COP1 binding sites (VP/AA) in CRTC2 on its ubiquitination by TRAF6 in HEK293T cells. (b) Effect of wild-type and VP/AA CRTC2 on *G6pase* mRNA amounts in hepatocytes exposed to IL-1β (10 μg/l) and glucagon (20nM). (*$p < 0.05$). (c) Immunoblots showing effect of COP1i on CRTC2 ubiquitination by TRAF6 in HEK293T cells. (d) Effect of unspecific RNAi (USi) and COP1 RNAi (COP1i) on *G6pase* mRNA amounts in hepatocytes exposed to IL-1β (10 μg/l) and glucagon (20nM). (*$p < 0.05$).
